# Cyclophosphamide arrhythmogenicitytesting using human-induced pluripotent stem cell-derived cardiomyocytes

**DOI:** 10.1038/s41598-020-79085-5

**Published:** 2021-01-27

**Authors:** A. D. Podgurskaya, M. M. Slotvitsky, V. A. Tsvelaya, S. R. Frolova, S. G. Romanova, V. A. Balashov, K. I. Agladze

**Affiliations:** 1 Moscow Institute of Physics and Technology (National Research University), Dolgoprudny, Moscow Region, 141701 Russian Federation; 2 M.F. Vladimirsky Moscow Regional Clinical Research Institute, Moscow, 129110 Russian Federation

**Keywords:** Toxicology, Heart stem cells

## Abstract

Cyclophosphamide (CP) is an anticancer drug, an alkylating agent. Cardiotoxicity of CP is associated with one of its metabolites, acrolein, and clinical cardiotoxicity manifestations are described for cases of taking CP in high doses. Nevertheless, modern arrhythmogenicity prediction assays in vitro include evaluation of beat rhythm and rate as well as suppression of cardiac late markers after acute exposure to CP, but not its metabolites. The mechanism of CP side effects when taken at low doses (i.e., < 100 mg/kg), especially at the cellular level, remains unclear. In this study conduction properties and cytoskeleton structure of human induced pluripotent stem cell-derived cardiomyocytes (hiPSC-CMs) obtained from a healthy donor under CP were evaluated. Arrhythmogenicity testing including characterization of 3 values: conduction velocity, maximum capture rate (MCR) measurements and number of occasions of re-entry on a standard linear obstacle was conducted and revealed MCR decrease of 25% ± 7% under CP. Also, conductivity area reduced by 34 ± 15%. No effect of CP on voltage-gated ion channels was found. Conduction changes (MCR and conductivity area decrease) are caused by exposure time-dependent alpha-actinin disruption detected both in hiPSC-CMs and neonatal ventricular cardiomyocytes in vitro. Deviation from the external stimulus frequency and appearance of non-conductive areas in cardiac tissue under CP is potentially arrhythmogenic and could develop arrhythmic effects in vivo.

## Introduction

The cardiotoxicity of anticancer therapy can cause life-threatening complications. In the last few decades, this topic has mainly been associated with anthracyclines. However, the use of other classes of anticancer drugs, including alkylating agents, can also result in serious heart disorders. Cyclophosphamide (CP), belonging to the class of alkylating agent, is one of the most effective and safe medicines essential in a health system. However, its application is often associated with atrial and ventricular tachyarrhythmias and complete atrio-ventricular block, hypotension, heart failure (HF; including congestive HF), and myocarditis (including hemorrhagic myopericarditis, leading to pericardial effusions, cardiac tamponade, and death)^1–6^. Manifestations of such side effects include a decreased amplitude of the QRS complex, non-specific T-wave or T-segment abnormalities, and asymptomatic left ventricular ejection fraction (LVEF) drop^1,4^. These disorders can be caused by a high dose of CP (i.e., 100–200 mg/kg)^1,3–5^. In addition, a past study reported fulminate fatal congestive heart failure occurring after a CP dose of 75 mg/kg was applied in a case of diffused large B cell lymphoma^7^—a rare clinical example of CP cardiotoxicity at a dose lower than 100 mg/kg. Acute symptoms may occur within 14 days after drug administration and can last up to 10 days after initiation^2,4–6^. However, in some patients (up to 10%), the symptoms may disappear spontaneously in 1–7 days^4,6^. The prevalence of fatal CP cardiomyopathy varies at between 2 and 17% of patients^1,4^. The pathomechanism involves the direct damage of endothelial cells and cardiomyocytes (CMs), interstitial hemorrhage, and edema. Autopsy studies showed the thickening of the left ventricular walls and interventricular septum, and the hemorrhagic necrosis of the myocardium. Due to the development of an intracapillary microemboli/coronary vasospasm under CP, it also causes ischemic damage^4,8^. In addition, the troponin I level was found to rise between 8 and 15 days after the administration of a high dose of CP, which may indicate direct myocardial damage^9^.

CP is an alkylating agent that undergoes hepatic metabolism and forms 4-hydroxy cyclophosphamide (4-HCY); 4-HCY metabolizes into aldophosphamide by CYP3A4/A5. Aldophosphamide, in turn, produces phosphoramide mustard, which is responsible for the anticancer activity, with further metabolism into the non-toxic compounds carboxyphosphamide and nitrogen mustard, along with acrolein. Acrolein is toxic and affects the myocardium and endothelial cells; however, CP itself is not associated with any cardiotoxic sequalae^8^. Less aldehyde dehydrogenases (ALDH) activity, increased reactive oxygen species (ROS) levels, and myocardial cytotoxicity were induced by 4-HCY and acrolein in H9c2 cells. In these cell cultures, 4-HCY was metabolized to acrolein^10^. Ultrastructural changes in rat CMs included the moderate lysis of myofibrils, the dilation of vesicles in the granular and agranular sarcoplasmic reticulum, and the destruction of mitochondria with the formation of myelin-like residual bodies after the intraperitoneal injection of CP in a single dose of 125 mg/kg^11^.

The lack of response to CP was found in a human CM arrhythmic risk model developed by Guo et al.^10^. This model was based on the detection of changes in the beat rhythm and rate of a confluent monolayer of human-induced pluripotent stem cell-derived cardiomyocytes (hiPSC-CMs; iCell Cardiomyocytes) caused by a compound, using real-time cellular impedance measurement. Arrhythmogenicity is quantified by predicted proarrhythmic score based on 2 concentrations of each drug: IB20, the lowest concentration at which ≥ 20% arrhythmic/irregular/atypical beats in 3 consecutive 20-s sweeps are induced; and BR20, the lowest concentration at which reduction in a spontaneous beat rate of ≥ 20% at 3 consecutive sweeps compared to the control is induced. This system allowed for the better prediction of torsadogenicity in humans in comparison to either human Ether-à-go-go-related gene (hERG) inhibition or QT prolongation assays. The other model that was used to test CP was human embryonic stem cell (hESC) model developed by Zhu et.al.^12^. It was based on hESC differentiation into cardiomyocytes in vitro with further distinction of cardiac precursor cells and late mature cardiomyocytes. IC50 value for CP toxicity towards mature cardiomyocytes was 476 ± 13 mg/l (1823 ± 50 µM). Nevertheless, data related to the cardiotoxicity of CP at concentrations less than 100 mg/kg^1^, as well as data on its influence on ion channels, are rare. As such, the mechanism of its action in producing arrhythmias is not fully understood.

In the current study, hiPSC cardiac tissue from a healthy donor was used to examine 3 values: the conduction velocity (CV), maximum capture rate (MCR), and number of occasions of re-entry on a standard linear obstacle after the application of 213–852 µM of CP using the optical mapping method. Earlier proposed arrhythmogenicity test using a standard linear obstacle developed by Slotvitsky M.M., Agladze K.I. et al.^13,14^ was validated. Patch-clamp experiments were conducted to measure currents through voltage-gated fast sodium (INav), delayed rectifier potassium (rapid: IKr; slow: IKs), and *L-type* calcium (ICaL) ion channels under CP. Immunocytochemistry was used to evaluate changes in the α-actinin structure in hiPSC-CMs under CP.

## Materials and methods

All studies were conducted by the National Institutes of Health Guide for the Care and Use of Laboratory Animals (NIH publications No. 8023, revised 1978) and approved by the Moscow Institute of Physics and Technology Life Science Center Provisional Animal Care and Research Procedures Committee, Protocol \#A2-2012-09-02.

### Fibroblast derivation and reprogramming to the pluripotent state, characterization of iPSC lines and differentiation of iPSCs into cardiomyocytes

Fibroblast derivation and reprogramming to the pluripotent state, characterization of iPSC line m34Sk3, differentiation of iPSCs into cardiomyocytes were performed as previously described in Materials and methods section in Ref.^14^. In brief, dermal fibroblasts isolated from a skin biopsy of healthy donor were nucleofected with episomal vectors expressed *OCT4*, *SOX2*, *KLF4*, *L-MYC*, and *LIN28* (Addgene IDs #41855-41858, #41813-41814). Surface for cell plating was coated with Geltrex LDEV-Free hESC-Qualified Reduced Growth Factor Basement Membrane Matrix. Reprogramming to the pluripotent state was performed as described in https://tools.thermofisher.com/content/sfs/manuals/epi5_episomal_ipsc_reprogramming_man.pdf. Spontaneous differentiation of the cell lines was carried out through embryoid bodies formation. iPSCs were cultivated for several passages under feeder-free conditions. Directed differentiation of iPSCs into cardiomyocytes was triggered by adding the RPMI 1640 medium (Lonza) contained B27 supplement minus insulin (Thermo Fisher Scientific) and 8 μM CHIR99021 (Sigma-Aldrich) for 48 h. The first cell contractions were observed from day 9 of differentiation. The flow cytometry data for cardiac markers of patient-specific iPSC-derived CMs from healthy donor that used in this study (m34Sk3 cell line) is provided in Ref.^15^, the efficiency of directed iPSC differentiation into cardiomyocytes was 47%. The differentiated cells were subjected to metabolic selection to isolate cardiomyocytes, almost all cells that passed the metabolic selection expressed cardiac troponin T on day 45. When the culture reached 50 days, the optical mapping occurred.

### Preparation of neonatal rat ventricular cardiomyocytes, preparation of samples

Preparation of NRVMs, preparation of samples were performed as previously described in Materials and methods section in Ref.^16^. In brief, isolation and seeding of NRVMs were performed according to the Worthington protocol (http://www.worthington-biochem.com/NCIS/default.html). For cell seeding and cultivation, 13- and 21-mm glass coverslips were covered with human fibronectin (Imtek) and placed in Petri dishes and 24-well culture plates. iPSCs were seated in the wells of 24-well sterile plates covered with Geltrex. The differentiation protocol started on day 3–4 after plating.

### Protocol of optical mapping

Optical mapping was carried out as previously described^13,14^. The setup included high-speed video camera (Andor IXon3, Andor Technologies), a mercury lamp (Olympus U-RFL-T), an optical microscope (Olympus MVX10), a filter cube (Olympus U-M49002XL), and an impulse generator (Vellemann, PCGU-1000), a platinum point electrode and a reference circular electrode.

Optical mapping of human iPSC-derived cardiomyocytes occurred 50 days after the start of differentiation protocol. Samples were incubated in a sterile medium at 37 °C with the fluorescent calcium-dependent Fluo-4 AM (Invitrogen, USA) dye in concentration 4 μg/ml for 30 min. Then the dye solution was exchanged with a sterile Tyrode’s solution (Sigma T2145) containing 136.9 mM NaCl, 2.6 mM KCl, 1.8 mM CaCl_2_, 1.1 mM MgCl_2_, 0.4 mM NaH_2_PO_4_, 11.9 mM NaHCO_3_, 5.6 mM d-glucose, pH = 7.4 with free Ca^2+^ concentration of 1.80 mM.

At first, the samples were checked for the presence of spontaneous beating. Then the passage of square pulses of 1 Hz frequency was initiated. If impulses of 1 Hz were captured by a tissue, the frequency of square pulses was increased in increment of ≤ 0.5 Hz. Each step was checked whether the impulses of set frequency are captured. When the tissue was not able to capture each of the external impulses (for example, 1:2 capturing) of set frequency, the value of frequency on the previous step was the MCR. After capturing the CV and MCR controls, the solution of CP (Baxter) in Tyrode’s was added to the cell culture at concentrations: 213 μM, 639 μM, 852 μM, 1065 μM in the increasing order. The CV and MCR were measured ≤ 10 min after the addition of CP solution. The CV was measured at a frequency of 1 Hz, for the MCR measurements the frequency was increased from 1 to 5 Hz in increments of ≤ 0.5 Hz. Three independent differentiations of m34Sk3 cell line were done with n = 2, n = 3 and n = 3 independent runs of experiments respectively. The experiments were carried out at 37 °C, 0.03–0.05% CO_2_.

As the metabolism of individual organism is specific, the correspondence between concentrations in vitro and concentrations in mg/kg could be estimated approximately. Setting the mass of the human body 60 kg and the blood volume of 4.8 L, 213–1065 µM CP in vitro correspond to 4.45–22.25 mg/kg.

### Standard linear obstacle

After adding Tyrode solution to the sample, a standard linear obstacle was made on the cell tissue perpendicular to the direction of excitation wave propagation (Supplementary Fig. 1). The obstacle width was ≤ 100 μm. The configuration of the propagating wave front under normal conditions and under various (213–852 μM) concentrations of CP in the presence of a standard linear obstacle was evaluated. N = 8 independent runs of experiments were conducted, and the number of occasions of re-entry formation were counted.

### Patch-clamp

Whole-cell currents were recorded using the perforated patch-clamp technique in single cardiomyocytes, which were isolated from neighboring cells. As a perforating agent, Amphotericin B in DMSO was used at a final concentration of 0.24 mg/ml^17^. A cover slip with cardiac cells was placed in the recording chamber mounted on the stage of the Olympus IX71 inverted microscope table. The pipette and the extracellular solutions used in these protocols are listed below.

The bathing solution used for recording Na^+^ current: 50 mM NaCl, 1.8 mM CaCl_2_, 1 mM MgCl_2_, 110 mM CsCl_2_, 10 mM d-glucose, 10 mM HEPES/NaOH (pH = 7.4 CsOH). The patch pipette was filled with a solution:135 mM CsCl_2_, 10 mM NaCl, 2 mM CaCl_2_, 5 mM EGTA, 10 mM HEPES/NaOH, 5 mM MgATP (pH = 7.2 CsOH). For recording Ca^2+^ current: 160 mM TEA-Cl, 5 mM CaCl_2_, 1 mM MgCl_2_, 10 mM d-glucose, 10 mM HEPES/NaOH (pH = 7.4 CsOH). The pipette solution contained 145 mM CsCl_2_, 5 mM NaCl, 5 mM EGTA, 10 mM HEPES/NaOH, 5 mM MgATP (pH = 7.2 CsOH). For the whole-cell recording of IKr currents, the bathing solution contained, 150 mM NaCl, 5.4 mM KCl, 1 mM MgCl_2_, 1.8 mM CaCl_2_, 15 mM d-Glucose, 15 mM HEPES/KOH (pH = 7.4 NaOH) and the patch pipette was filled with a solution containing 150 mM KCl, 2 mM CaCl_2_, 5 mM NaCl, 5 mM MgATP, 5 mM EGTA, 10 mM HEPES/KOH (pH = 7.2 KOH). Nifedipine was used to block calcium channels^18^.

Voltage clamp experiments were performed as previously described in Materials and Methods section in Ref.^19^. Patch pipettes were pulled from borosilicate glass (BF150-86-10 Sutter Instrument, USA) with tip resistances of ~ 3 MΩ when placed into the experimental solution. The pipette offset was corrected to zero just prior to the formation of a gigaohm (GΩ) seal. After formation of the GΩ seal, the pipette capacitance was cancelled using the amplifier fast capacitance cancellation settings. Electrical access to the cell by perforation was indicated by the appearance of slow capacitance currents that increased the amplitude and rate of decay when more amphotericin pores formed in the membrane enclosed by the patch pipette. The access resistance was monitored using the slow whole-cell capacitance cancellation settings on the amplifier. Once the access resistance decreased below 12 MΩ, the experiment was started. Series resistance was compensated if required.

Whole-cell currents evoked by ramping up stimuli from − 120 to + 50 mV was examined over a 200-ms period, with a holding potential (HP) of − 80 mV (using a prestep: − 80 to − 120 mV for 100 ms)^20^. The voltage-dependence of the peak Na^+^ currents was determined by measuring peak inward currents for cells depolarized from − 80 to + 15 mV in 5-mV increments, which were applied for 200 ms. To detect *L-type* Ca^2+^ currents without contamination from Na^+^ currents, a 100-ms prepulse to − 40 mV from a HP of − 80 mV was used^21,22^. The peak ICaL was measured at 0 mV. Outward IKr was elicited by a 5-s depolarizing pulse from − 40 mV to + 50 mV in 10-mV increments (HP of − 40 mV). IKr was isolated as an E4031-sensitive current^23^. Typically, the membrane capacitances measured with pCLAMP10.2 software ranged from 20 to 50 pF.

The experiments were carried out at 37 °C, 0.03–0.05% CO_2_.

### Immunofluorescent staining

The protocol used for fixation and immunocytochemistry of the samples was made due to recommendations from https://www.abcam.com/protocols/immunocytochemistry-immunofluorescence-protocol. Immunofluorescent staining was performed as previously described^13^. Cells were fixed for 10 min in 4% paraformaldehyde, permeabilized for 10 min in 0.4% Triton-X100. Cells were further incubated for 30 min in blocking buffer (1% bovine serum albumin in phosphate-buffered saline, PBS), overnight at 4C with primary antibodies and for 1 h at room temperature with secondary antibodies. Cells were washed twice for 15 min in PBS. Nuclei were stained with DAPI. Primary antibodies (working dilution—1:100)—sarcomeric alpha-actinin (Abcam, ab9465). Secondary antibodies (Thermo Fisher Scientific, working dilution—1:400)—Alexa Fluor 568 goat anti-mouse IgG (HþL) highly cross adsorbed (A11031). Alexa Fluor 488 phalloidin (Molecular Probes, USA, A12379) was used for F-actin non-specific staining.

### Assessment of the structural integrity of the hiPSC-CMs cytoskeleton

Normally, α-actinin in cardiomyocytes is associated with F-actin, which is organized in several parallel bundles. These structures are greatly affected by CP. The degree of this effect was estimated using the Fast Fourier Transform and Directionality plugin in ImageJ (NIH, Maryland, USA, http://rsb.info.nih.gov/ij) software. It allowed to analyze a distribution of structures orientation present in the input image.

F-actin parallel bundles cross-linked by α-actinin appear in the distribution charts as peak values compared to the baseline intensity. Damage of cellular structures leads to a decrease in peak intensities and an increase in baseline intensity. The contrast of the highest peak was calculated according to Weber's definition^24^ as:

$${\text{C}} = ({\text{Imax}} - {\text{Ibl}})/{\text{Ibl,}}$$

where C denotes contrast; Imax is the maximal preferred structures orientation; Ibl is the baseline intensity calculated by Directionality plugin.

### Data processing and statistics

Data processing was performed as previously described^16^. All videos from optical mapping and images from the confocal microscope were processed in the ImageJ. The activation maps were built using Wolfram Mathematica. The statistical significance was determined using a one-way ANOVA followed by Fisher's least significant difference for comparisons among groups. Values of *p* < 0.05 were considered statistically significant.

## Results

### Optical mapping of hiPSC-CMs with CP

**Figure 1:**
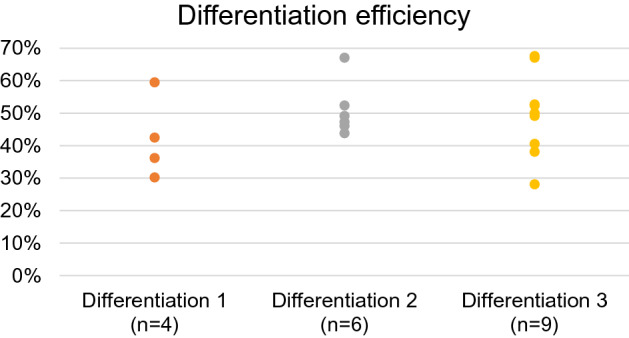
Human iPSC-CMs differentiation efficiency for three independent differentiations of m34Sk3 cell line. Quantification was done on the basis of optical mapping fluorescence data (Ca^2+^) of obtained cardiac tissue.

In the first set of experiments, efficiency assessment of three differentiations that were performed in this study was done on the basis of optical mapping fluorescence data. Using Fluo-4 dying of obtained cardiac tissue, amplitude maps were constructed and the percent of cardiomyocytes was calculated. The percentage of CMs in the tissue, and, therefore, average efficiency of each of three independent differentiations was 42% (n = 4), 51% (n = 6) and 50% (n = 9) (Fig. 1).

**Figure 2:**
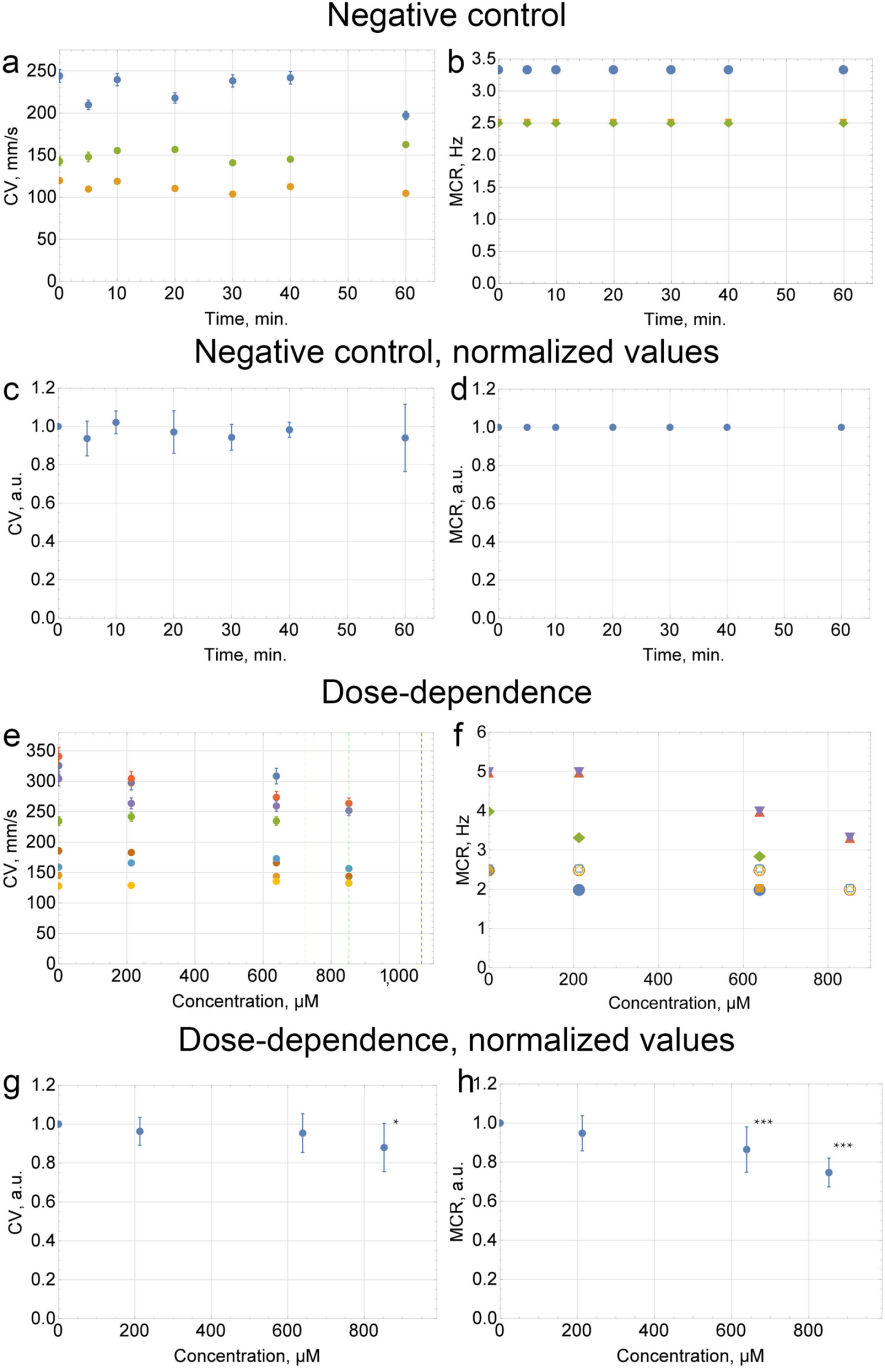
The CP dose-dependences of CV and MCR in the hiPSC cardiac tissue. The CV was measured at a frequency of 1 Hz. For the MCR measurements, the frequency was increased from 1 to 5 Hz in increments of ≤ 0.5 Hz. Dashed lines indicate the moments when normal propagation stopped. The CV and MCR were measured ≤ 10 min after the addition of the CP solution. (**c**,**d**,**g**,**h**) Data of each run was normalized to the control value. Summary data is presented as mean ± SD (n = 8); *p < 0.05, **p < 0.01, ***p < 0.001 vs. control. (**a**,**e**) Error bars on each individual point represent the equipment error for each measurement of CV.

In the second set of experiments, the CP dose-dependences of the conduction velocity (CV) and maximum capture rate (MCR) were measured in the hiPSC cardiac tissue. Three independent differentiations of m34Sk3 cell line were done with n = 2, n = 3 and n = 3 independent runs of experiments. Negative control data of CV (Fig. 2a,c) and MCR (Fig. 2b,d) was obtained during incubation of the cells with Tyrode’s solution. In 1 of the 8 runs of experiments, normal propagation stopped after the addition of 724 µM (~ 15 mg/kg) of CP, and in another 2 runs after the addition of 852 µM (~ 18 mg/kg). In the other 5 runs normal propagation stopped after the addition of 1065 µM (~ 22 mg/kg) of CP (Fig. 2e). Figure 2f illustrates MCR values for each run. No considerable difference in CV was found during CP treatment (Fig. 2g) up to 852 µM (~ 18 mg/kg). The MCR was stable within the margin of error after the addition of 213 µM (~ 4 mg/kg) of CP. The MCR fell by 25% ± 7% in comparison to the control values after the addition of 852 µM (~ 18 mg/kg) of CP (Fig. 2h).

**Figure 3:**
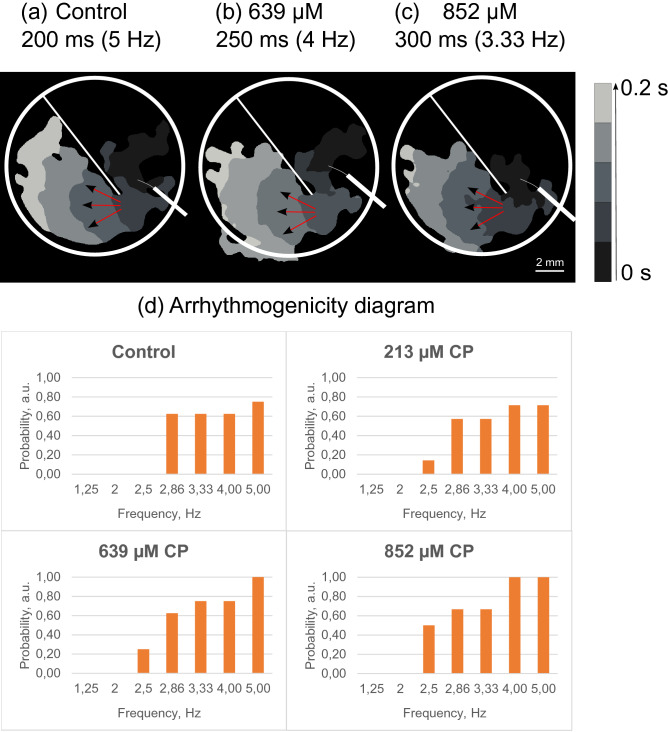
The effect of cyclophosphamide on re-entry formation in the hiPSC cardiac tissue. Activation maps of successful propagation on a standard linear obstacle in (**a**) control, (**b**) under 639 µM (~ 13 mg/kg) and (**c**) 852 µM (~ 18 mg/kg) of CP. The activation time is color coded. The frequency was increased from 1 to 5 Hz in increments of ≤ 0.5 Hz; white circles correspond to the border of the sample, white lines correspond to the location of the obstacle, and red arrows indicate the direction of the excitation wave propagation. Arrhythmogenicity diagram (**d**), the orange bars indicate cases in which the tissue did not capture the external impulses. The vertical values of the diagram bars show the probability of the external impulses non-capturing in the hiPSC-derived cardiac tissue.

In the third set of experiments, re-entry occurrence was checked on a standard linear obstacle in hiPSC cardiac tissue (n = 8) after the addition of 213 µM (~ 4 mg/kg), 639 µM (~ 13 mg/kg), or 852 µM (~ 18 mg/kg) of CP. While increasing the frequency of the external impulses from 1 to 5 Hz in increments of ≤ 0.5 Hz, no re-entry was observed (Fig. 3).

However, arrhythmogenicity diagram on Fig. 3d illustrates the increase of non-capturing probability with the increase of CP dose. The impulses of the highest frequencies captured in control (4 Hz and 5 Hz) were not captured in all of the samples under 852 µM CP.

The conductivity area of excitation wave propagation at different doses of CP was measured. Setting the conductivity area in control as 100%, conductivity areas were 92 ± 8% (n = 8, p < 0.01 vs control), 82 ± 10% (n = 8, p < 0.001 vs control) and 66 ± 15% (n = 8, p < 0.01 vs control) under the influence 639 µM (~ 13 mg/kg), 852 µM (~ 18 mg/kg) and 1065 µM (~ 22 mg/kg) of CP respectively.

### Patch-clamp experiments on hiPSC-CMs with CP

**Figure 4:**
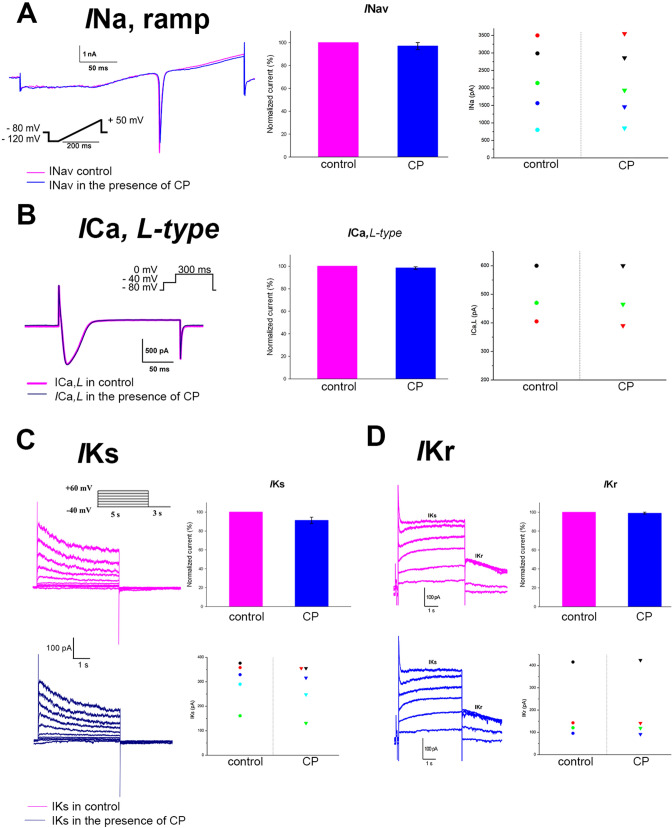
The effect of CP on voltage-dependent ion channels in hiPSC-CMs obtained from a healthy donor. (**a**) Voltage-dependent fast sodium current (INav) shown in the control and after the addition of 630 μM (~ 13 mg/kg) of CP, and a ramp current that was evoked when the voltage was increased smoothly from − 120 to + 50 mV for 200 ms. The cell was prepulsed to − 120 mV for 100 ms from a holding potential of − 80 mV. The voltage protocol is shown above the current trace. Summary data is presented on the bar graph as mean ± SEM (n = 5). (**b**) *L-type* Ca^2+^ currents obtained in the absence (control) and presence of 630 μM (~ 13 mg/kg) of CP. Inset: The original current trace in response to a voltage step from − 40 to 0 mV for 300 ms. The inactivation of INa^+^ was achieved by a pre-step from a holding potential of − 80 mV to − 40 mV for 100 ms. Summary data is presented on the bar graph as mean ± SEM (n = 3). (**c**) The effect of 630 μM (~ 13 mg/kg) of CP on the slow potassium currents of the delayed rectification IKs current elicited in response to the 5 s depolarizing pulse from − 40 mV to + 60 mV in 10 mV increments. Summary data is presented on the bar graph as mean ± SEM (n = 5). (**d**) Voltage-clamp recordings of the IKr current elicited in response to the 5 s depolarizing pulse from − 40 mV to + 60 mV in 10 mV increments (HP of − 40 mV), showing the effect of 630 μM (~ 13 mg/kg) of CP on a tail current of IKr. IKr tail after the stimulation step during a 3 s holding potential of − 40 mV could be observed. Summary data is presented on the bar graph as mean ± SEM (n = 4).

Patch-clamp experiments were conducted on isolated hiPSC-CMs obtained from a healthy donor. According to the results of experiments, voltage-dependent INav, ICaL, IKs and IKr ion currents were detected in cardiomyocytes without changes in the presence of 630 μM (~ 13 mg/kg) CP with an experiment duration of no more than 30 min (Fig. 4).

The ion currents of voltage-dependent ion channels were fixed unchanged after 5, 10, 15, 20, 25 min after the start of exposure to CP. After 30 min and more, vacuoles appear in isolated cells, the cells begin to die by visual observation under a microscope in the presence of 630 μM (~ 13 mg/kg) CP.

### Optical mapping of neonatal rat ventricular myocytes (NRVMs) with CP

**Figure 5:**
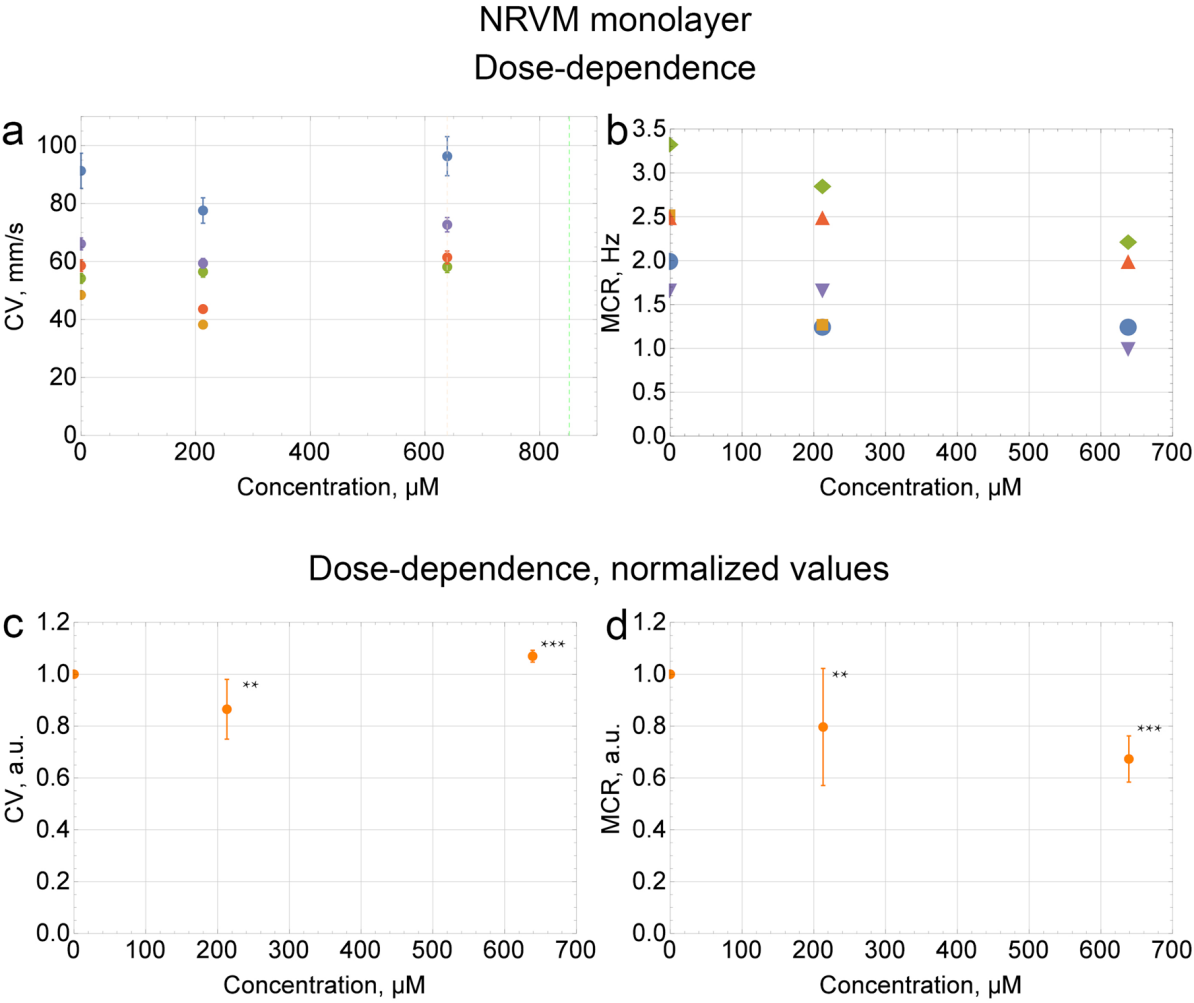
The CP dose-dependences of CV and MCR in the NRVM monolayer. The CV was measured at a frequency of 1 Hz. For the MCR measurements, the frequency was increased from 1 to 5 Hz in increments of ≤ 0.5 Hz. Dashed lines indicate the moments when normal propagation stopped. (**a**) Error bars on each individual point represent the equipment error for each measurement of CV. (**c**,**d**) Data of each run was normalized to the control value. Summary data is presented as mean ± SD (n = 5); **p < 0.01, ***p < 0.001 vs. control.

The CP dose-dependences of CV and MCR were measured in neonatal rat ventricular myocytes (NRVM) monolayers. In 1 of the 5 runs of experiments, normal propagation stopped after the addition of 639 µM (~ 13 mg/kg) of CP, and in another 4 runs after the addition of 852 µM (~ 18 mg/kg) (Fig. 5a). Figure 5b illustrates MCR values for each run. No considerable difference in CV was found during CP treatment (Fig. 5c) up to 639 µM (~ 13 mg/kg). The MCR was stable within the margin of error after the addition of 213 µM (~ 4 mg/kg) of CP. The MCR fell by 33% ± 9% in comparison to the control values after the addition of 639 µM (~ 13 mg/kg) of CP (Fig. 5d). The conductivity area of excitation wave at 852 µM (~ 18 mg/kg) of CP was 77 ± 13% (n = 5, p < 0.05 vs control).

### Immunocytochemistry of hiPSC-CMs and NRVMs

**Figure 6:**
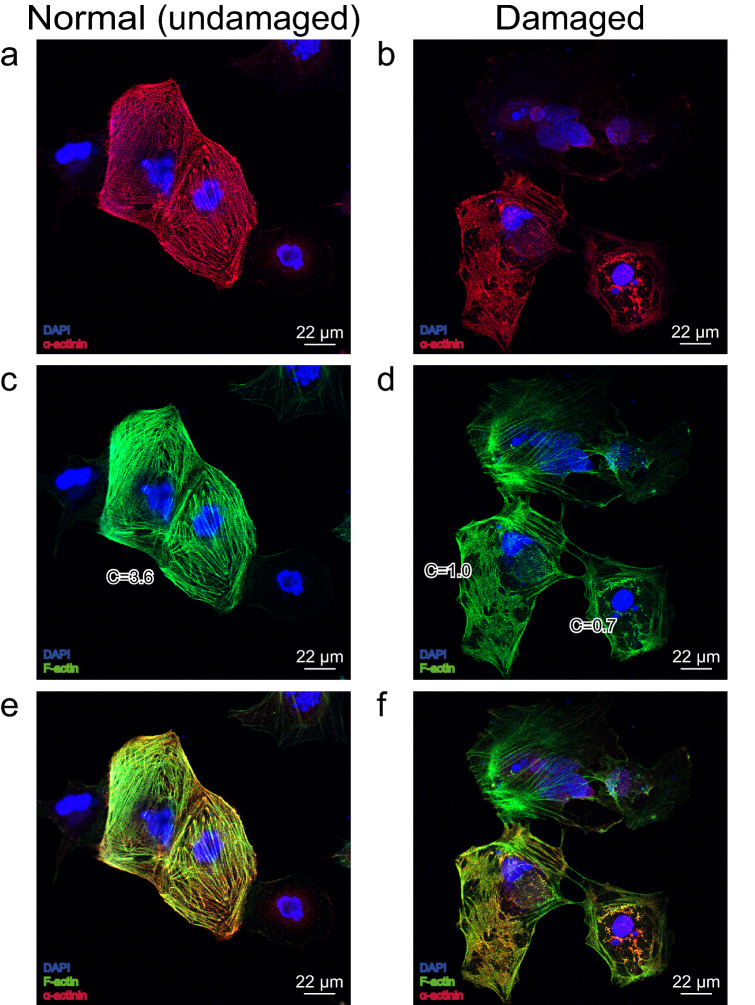
Confocal imaging of F-actin, α-actinin and DAPI in hiPSC-CMs. (**a**,**c**,**e**) Normal, undamaged cardiomyocytes. C = 3.6 for the presented cells. (**b**,**d**,**f**) Bottom part of figure: cardiomyocytes with damaged cytoskeleton after incubation with 213 µM (~ 4 mg/kg) of CP. C = 1.0 and C = 0.7 for cardiomyocytes presented on the image.

Samples with ~ 50 hiPSC-CMs (both isolated and in clusters) were prepared. Control samples and samples after 5, 10, 20, and 30 min incubation with 213 µM (~ 4 mg/kg) of CP were fixated and further stained for F-actin, α-actinin, and DAPI (Fig. 6a–f). Modification of the α-actinin structure under CP was found (Fig. 6b, Table 1). Distribution of structures orientation present in the image is characterized as C-value (calculation of the C-value is described in Materials and Methods section, paragraph 7). C-value for normal, undamaged cardiomyocytes that were presented in control samples, was 1.53 ± 0.67 (n = 9) and for cardiomyocytes with damaged cytoskeleton after 30 min incubation with 213 µM (~ 4 mg/kg) of CP—0.64 ± 0.18 (n = 9, p < 0.001 vs control).

**Table 1:** The influence of CP on hiPSC-CM and NRVM cytoskeleton in relation to incubation time.

Incubation with CP	hiPSC-CMs: n_undamaged_/n_total_	NRVMs: n_undamaged_/n_total_
0 min	0.82	0.55
5 min	0.75	0.43
10 min	0.48	0.27
20 min	0.34	0.3
30 min	0.42	0.27

**Figure 7:**
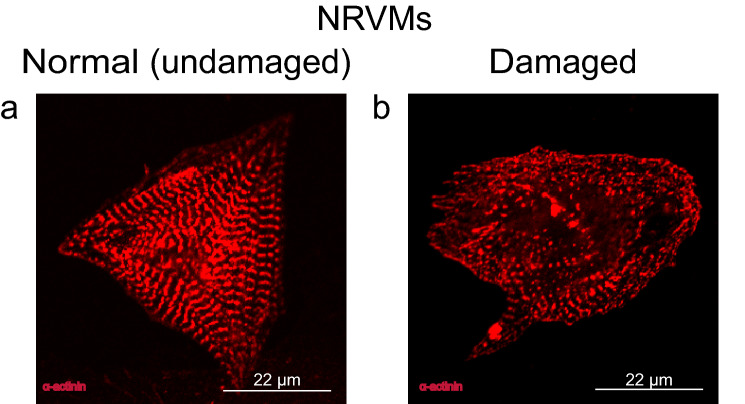
Representative image of α-actinin damage in isolated NRVMs under the influence of CP. (**a**) Normal, undamaged cardiomyocyte. (**b**) Cardiomyocyte with damaged α-actinin after incubation with 213 µM (~ 4 mg/kg) of CP.

Samples with ~ 100 isolated of NRVMs were prepared. Control samples and samples taken after 5, 10, 20, and 30 min of incubation with 213 µM (~ 4 mg/kg) of CP were fixated and further stained for α-actinin. Modification of the α-actinin structure under CP was also found (Fig. 7).

The proportion of CMs with a normal cytoskeleton (i.e., “undamaged”) in relation to the total number of CMs per sample was calculated for each time interval of incubation with CP (Table 1). For hiPSC-CMs samples, the number of “undamaged” isolated CMs and “undamaged” CM clusters were summarized, and the proportion to the total number of isolated CMs and CM clusters for each time interval was calculated. The boundary time at which the number of undamaged cells was close to that of the control (a 9% and 22% decrease for hiPSC-CMs and NRVMs, respectively) was 5 min.

## Discussion

CP is an anti-cancer drug that is used in the treatment of rheumatoid arthritis, lupus erythematosus, multiple sclerosis, neuroblastoma, and other types of cancer and it is also used in transplantology^25^. Unfortunately, its action is often associated with cardiovascular side effects such as atrioventricular block, tachyarrhythmias, heart failure, and myocarditis when taken in high doses (i.e., 100–200 mg/kg)^1–6^. While heart failure and myocarditis are caused by metabolites of CP^8^, atrioventricular block and tachyarrhythmias might be associated with the mechanisms of arrhythmia occurrence under CP influence on a cellular level, particularly when small doses of the drug are taken (i.e., less than 100 mg/kg).

To directly test the arrhythmogenic properties of CP and not its metabolites, we used an experimental model based on CMs obtained from the induced pluripotent stem cells of a healthy donor. 3 values were measured: the conduction velocity (CV), maximum capture rate (MCR), and number of occasions of re-entry on a standard linear obstacle (Supplementary Fig. 1) after the application of CP using the optical mapping method. The obtained 3 values compose the arrhythmogenicity test previously published by the authors^13,14^. Electrophysiological parameters (action potential duration APD, stable value of CV, the response to periodic stimulation in the range of physiological values etc.) of these cells were measured by Slotvitsky M.M., Agladze K.I. et al. previously^26^ and reached the same values as in mature cardiomyocytes at the 50th day of differentiation. As a spontaneous activity could be either a limit cycle or stochastic parameter depends on stochastic gating of transmembrane currents and of calcium release channels, it was not investigated in this study^27,28^.

The CV was stable within the margin of error under 213–852 µM (~ 4–18 mg/kg) CP. The level of Cx43 in a cardiomyocyte tissue/monolayer is connected with changes of conduction velocity. The significant reduction of intercellular coupling is required to cause minor slowing of conduction velocity^29,30^. Recent study provided the dependence of CV on Cx43 level in strands of ventricular myocytes. The average value of the CV was > 1.5 times slower in cell strands combining 70% wild type cells and 30% Cx43 knock out cells vs 100% wild type cell samples (p < 0.05). 50% wild type cells and 50% Cx43 knock out cells combination showed CV reduce > 5 times vs 100% wild type cell samples (p < 0.05)^31^ and the conduction block. Thus, the fact that conduction velocity was stable within the margin of error after applying CP in this study leads to the conclusion that intercellular contacts might not be influenced by 213–852 µM (~ 4–18 mg/kg) of CP.

However, in this study, a decrease in the MCR of the hiPSC cardiac tissue of up to 25% ± 7% under the influence of 852 μM (~ 18 mg/kg) was observed. The conductivity area of excitation wave was dose-dependently reduced and the normal propagation has stopped in all of the samples under 1065 µM (~ 22 mg/kg). The negative effect of CP manifested in maximum capture rate and conductivity area decrease was also found in NRVMs. These effects could be induced either by a disruption in the functioning of ion channels or damage to the cell structures’ integrity.

Our study shows that CP has no effect on the voltage-dependent ICaL, IKs and IKr, and INa ion channels (Fig. 4) in the concentration of 630 μM (~ 13 mg/kg) with an experiment duration ≤ 30 min. Nevertheless, CP caused exposure time-dependent changes in the α-actinin structure in the part of the seeded CMs (Table 1). It was detected both in human iPSC-CMs from a healthy donor and in NRVMs. Moreover, detachment process of the cells’ edge occurred after 30 min of incubation with CP (see SEM micrographs of hiPSC-CMs in Supplementary Fig. 3). Such structural changes may lead to the reduction of the conductivity area and cause the MCR decrease under the influence of CP. This is consistent with the fact that clinical QT prolongation may also be a consequence of structural injury to the myocardium^32^. Thus, a possible mechanism of the CP effect on CMs in vitro is shown to be related to the structural injury of the cells rather than to effects on the voltage-gated ion channels.

As shown in previous works, CP showed no response according to the hiPSC-CM (iCell Cardiomyocytes) arrhythmic risk model developed by Guo et al.^33^ based on real-time cellular impedance measurement. The absence of re-entry on a standard linear obstacle in this study proves these results. Re-entry formation presence under CP was checked on the whole range of frequencies of the external impulses from 1 to 5 Hz in increments of ≤ 0.5 Hz. No re-entry on the standard linear obstacle in hiPSC cardiac tissue was observed during CP treatment. Three drugs with confirmed proarrhythmic risk in clinic and known effects on voltage-dependent ion channels were used as the positive control (experiments were conducted on the current experimental setup) and published before^13,14^. Lidocaine and E-4031 were tested on patient-specific hiPSC cardiac tissue from a healthy donor (one cell line, ISMA6L)^13^. Lidocaine, fast sodium channel blocker, at the concentration 100 µM induced re-entry formation on the sharp end of the obstacle at 2 Hz, higher than 2 Hz frequencies of external stimulation were not captured by the tissue. E-4031, hERG-channel blocker, in a concentration of 1.6 µM induced re-entry formation on the sharp end of the obstacle at 1.7 Hz and lower frequencies of the external impulses, higher than 1.7 Hz frequencies were not captured by the tissue. Erythromycin was tested on the same model used in this study, patient-specific hiPSC cardiac tissue from a healthy donor, cell line m34Sk3^14^. Erythromycin, IKr channel blocker, at the concentrations 15–30 µM induced re-entry formation on the sharp end of the obstacle at 2–2.5 Hz, higher frequencies of external stimulation were not captured by the tissue. As discussed above, the re-entry formation is connected with voltage-gated ion channel blockage, which was not found during CP treatment. Arrhythmogenicity diagram on Fig. 3d illustrates that frequencies higher than 4 Hz were not captured by the tissue under 639 µM (~ 13 mg/kg) CP and frequencies higher than 3,33 Hz were not captured by the tissue under 852 µM (~ 18 mg/kg) CP, whereas 4 Hz and 5 Hz were captured in control. Deviation from the external stimulus frequency could be regarded as arrhythmic beats and should be considered when using CP for disease treatment. Moreover, appearance of non-conductive areas in cardiac tissue under CP could develop arrhythmia in vivo.

## Limitations

iPSC-CMs are often regarded as immature electrophysiologically^34,35^. Nevertherless, in this research tissue of 50 days old human iPSC-CMs were used for cardiotoxicity testing. Electrophysiological characteristics of these cells were measured previously^26^ and reached the same values as in mature cardiomyocytes after the 50th day of differentiation**.**

Selected patient-specific iPSC line (m34Sk3 line) from healthy donor is well studied by authors in all aspects related to this work: the structure of hiPSC-CMs tissue^26^, maturation^26^, reference tests for arrhythmogenicity with hERG channels blockage^14,26^, studies of electrophysiology and ion channels (E4031)^26^. The selected differentiation protocol was optimized and published earlier^14^. Stable differentiation efficiency was obtained^15,26^ (Fig. 1). However, since one line of patient-specific human iPSC (m34Sk3) is used in this work, each particular conclusion cannot be generalized to other cell lines of human iPSC-CMs.

### Compliance with ethical standards

The cell line is provided by the E. Meshalkin Novosibirsk Scientific Research Institute of Circulation Pathology and handling approved by the Institute of Circulation Pathology Ethics Committee (#27, March 21, 2013). The generation of iPSC line from cells donated by patient with informed consent described in Refs.^14,15^. All experiments and procedures were performed in accordance with principles for human experimentation as defined in the 1964 Declaration of Helsinki and its later amendments and were approved by the Scientific Council of the MIPT Life Science Center.

All applicable international, national, and/or institutional guidelines for the care and use of cell lines were followed.

## Conclusions

This study examined the conduction properties and cytoskeleton structure of hiPSC cardiac cells (m34Sk3 patient-specific cell line) obtained from a healthy donor under low doses (i.e., < 100 mg/kg) of CP. CP has distinct negative effect on cardiac tissue in vitro which is manifested in the reduction of maximum capture rate (the maximum rate at which each stimulus from the electrode was followed by a response) and the conductivity area.

Maximum capture rate decreased up to 25% ± 7% after application of 852 µM (~ 18 mg/kg) cyclophosphamide for ≤ 10 min. The conductivity area of excitation wave was 66 ± 15% under the influence 1065 µM (~ 22 mg/kg) of CP for ≤ 10 min. CP has no direct effect on re-entry formation, measured according earlier proposed method of cardiac tissue optical mapping^13,14^. It could be explained by absence of effect on voltage-gated ion channels in patch-clamp experiments in this study on hiPSC cardiac cells (m34Sk3 patient-specific cell line) obtained from a healthy donor. Immunocytochemical labeling revealed an exposure time-dependent disruption of α-actinin, which indicated the CP influence on the cardiomyocytes structure and, consequently, on conduction properties of the cardiac tissue. Thus, CP arrhythmogenicity testing showed deviation from the external stimulus frequency under 639 µM (~ 13 mg/kg) and 852 µM (~ 18 mg/kg) CP and appearance of non-conductive areas in cardiac tissue, which is potentially arrhythmogenic and could develop arrhythmic effects in vivo.

## Supplementary Information

Supplementary Information.

## Data Availability

The datasets generated and analysed during the current study, plugins for data processing are available from the corresponding author on reasonable request.
