# Supplementary material for: Cyclophosphamide arrhythmogenicitytesting using human-induced pluripotent stem cell-derived cardiomyocytes
Source: Sci Rep. 2021 Jan 27;11:2336. doi: 10.1038/s41598-020-79085-5 (PMC7841168; doi:10.1038/s41598-020-79085-5)
Supplement: Supplementary file 1 — Supplementary Information. [file 41598_2020_79085_MOESM1_ESM.docx]

**Cyclophosphamide arrhythmogenicity testing using human-induced pluripotent stem cell-derived cardiomyocytes**

Podgurskaya A.D.^1^, Slotvitsky M.M.^1^, Tsvelaya V.A.^1^, Frolova S.R.^1^, Romanova S.G.^1^, Balashov V.A.^1^, Agladze K.I.^1*^

1. Moscow Institute of Physics and Technology, Moscow, Russia

^*^Corresponding author: [agladze@yahoo.com](mailto:agladze@yahoo.com), +7 (965) 403-26-37, +7 (495) 408-79-96

**Supplementary materials**


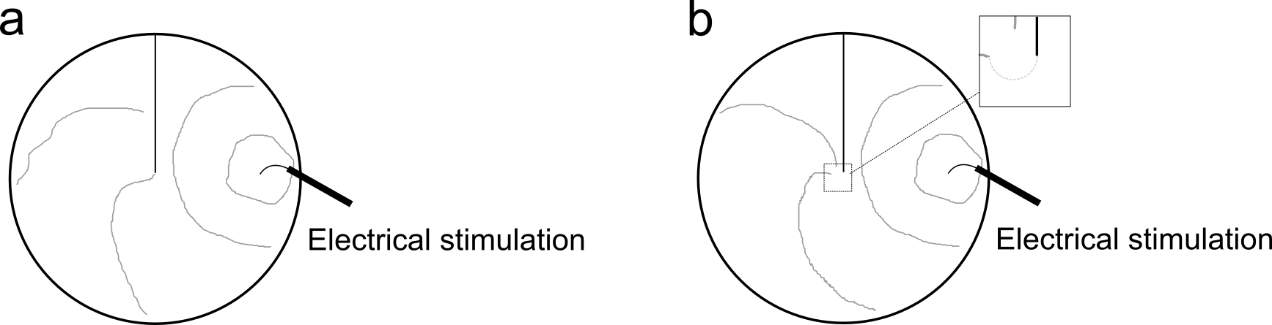


**Supplementary Figure 1.** The principal scheme of re-entry formation on the standard linear obstacle in the cardiac tissue *in vitro*. Black circle corresponds to the border of the sample (i.e., a Petri dish), black line corresponds to the unexcitable obstacle, grey curves indicate the excitation wave propagation front. (a) Successful propagation around a standard linear obstacle without re-entry formation; (b) The excitation wave front breaks on the obstacle and forms re-entry.

**
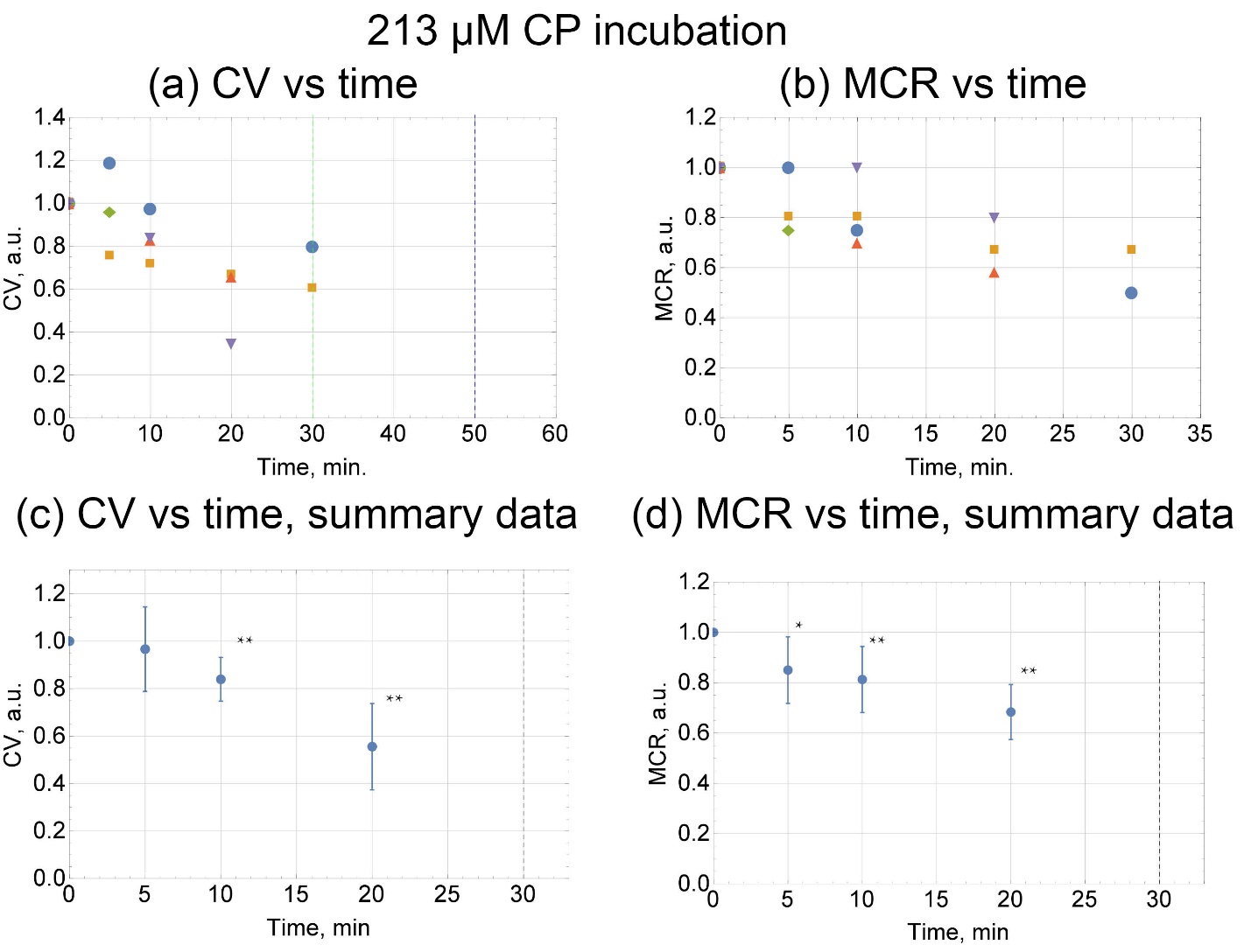
**

**Supplementary Figure 2.** The effect of 213 µM (~4 mg/kg) of CP on CV and MCR in the hiPSC cardiac tissue (n = 5). The CV was measured at a frequency of 1 Hz. For the MCR measurements, the frequency was increased from 1 Hz to 5 Hz in increments of ≤ 0.5 Hz. (a) The dashed lines indicates the moment when normal propagation stopped. (c),(d) The dashed line indicates the moment when normal propagation stopped in most of the samples. Data of each run was normalized to the control value. Summary data is presented as mean ± standard deviation (SD); *p < 0.05, **p < 0.01 vs. control.

CV and MCR were measured at 5, 10, 20, 30 and 50 min after the addition of 213 µM (~4 mg/kg) of CP to the hiPSC cardiac tissue. After 30 min of incubation, normal propagation stopped in 3 of 5 runs (Supplementary Fig. 2a, green dashed line). In the other 2 runs normal propagation stopped after 50 min of incubation (Supplementary Fig. 2a, blue dashed line).

***Scanning electron microscopy***

Materials and methods

In preparing the sample for scanning electron microscopy the classical air-drying method was used ^1^. Briefly, cardiac cultures on coverslips from control and experimental groups were double fixed in 2.5% glutaraldehyde on phosphate-buffered saline and 4% osmium tetroxide on water. After that the samples were dehydrated in graded aqueous ethanol solutions (70%, 80%, 90%, 97%) and absolute ethanol. Then the samples were plunged into hexamethyldisilane for 30 minutes and air-dried for 1 hour. To make their surface conductive they were sputter coated with 10 nm gold layer. The scanning electron microscopy (SEM) micrographs were done with JEOL JSM-6510LA microscope.
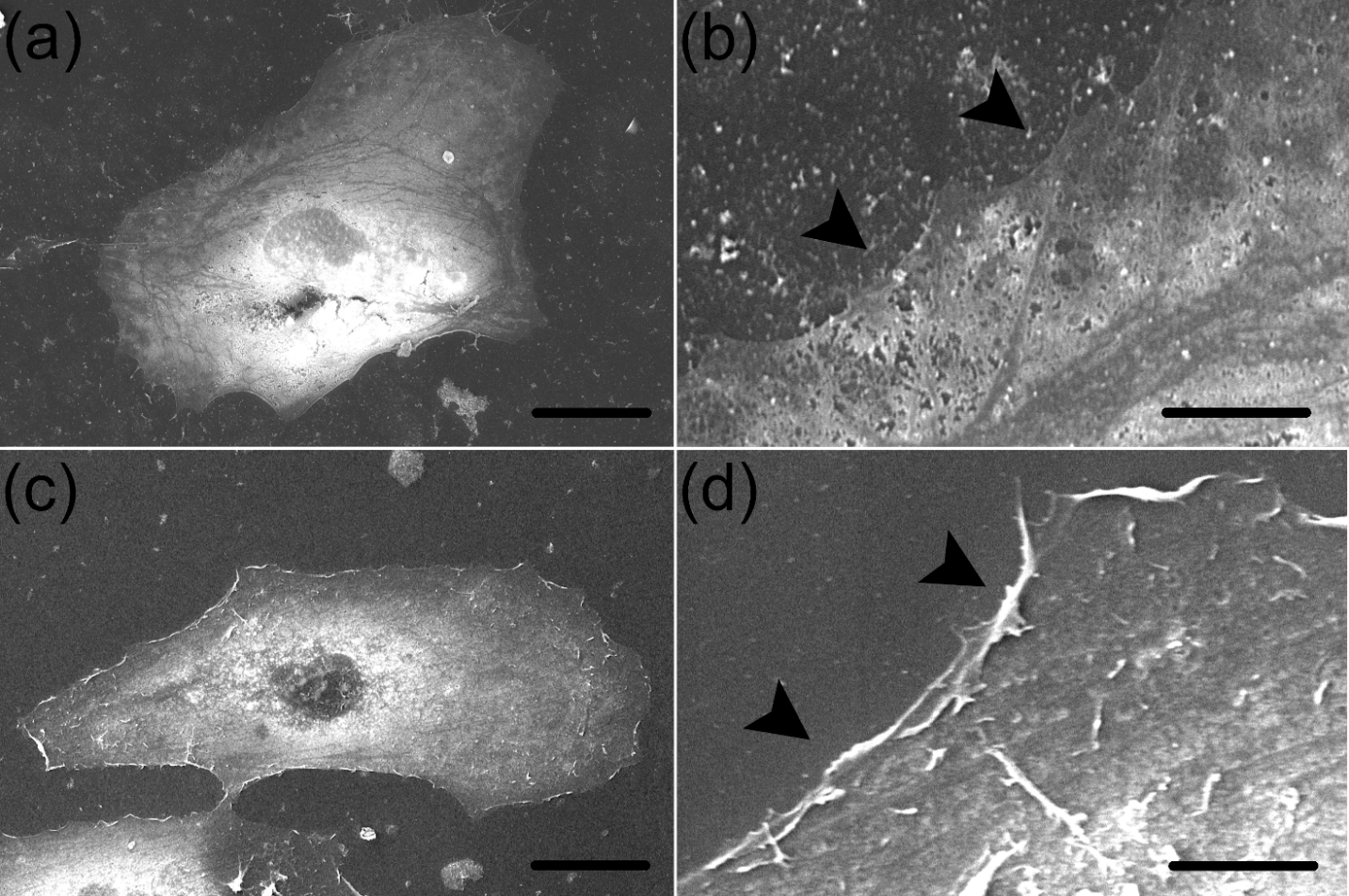
**Supplementary Figure 3.** **SEM micrograph of hiPSC-CMs from healthy donor (m34Sk3 line) on coverslips in control and after treatment with CP for 30 minutes.** (a) A SEM image of a typical cardiomyocyte on a flat glass substrate. Scale bar 30 µm. (b) The magnified image of the cell’s edge that fully adhere to the substrate. Scale bar 6 µm. (c) The micrograph of a cardiomyocyte after treatment with CP for 30 minutes. Scale bar 30 µm. (d) The magnified image of the cell’s edge after treatment with CP for 30 minutes. Black arrows indicate the cell’s regions where detachment process occurred. Scale bar 6 µm.

*Patch-clamp*

*
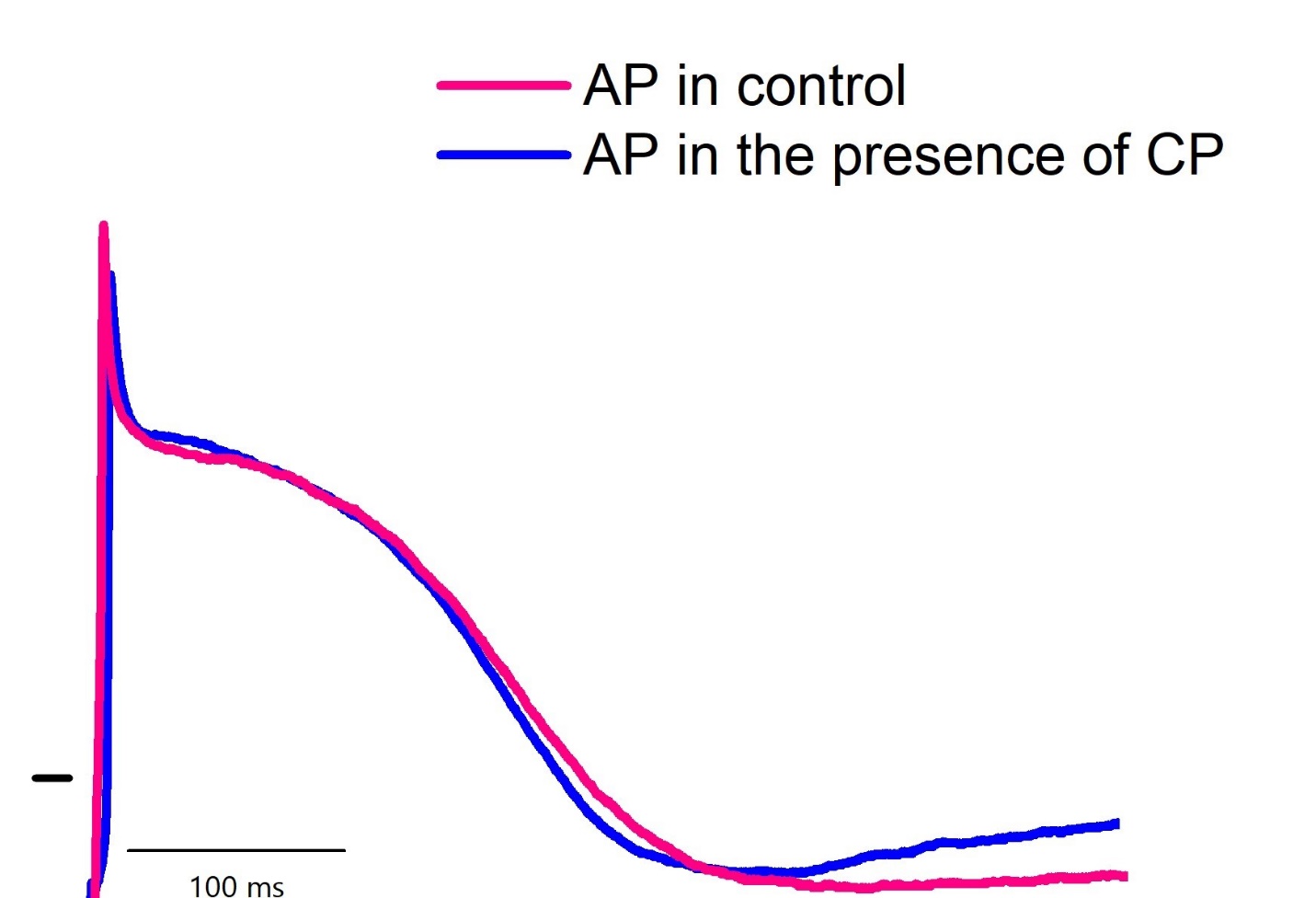
*

**Supplementary Figure 4. Action potential of hiPSC-CMs from healthy donor (m34Sk3 line) in control (pink) and in the presence of 630 μM cyclophosphamide (blue).** A form and a duration of the AP was not changed under the cyclophosphamide.

**References**

1. Pogorelov, A. G. & Selezneva, I. I. Evaluation of collagen gel microstructure by scanning electron microscopy. *Bull. Exp. Biol. Med.* **150**, 153–156 (2010).
